# Multimodal cell death drives the immunopathogenesis of RSV infection

**DOI:** 10.3389/fimmu.2025.1740904

**Published:** 2025-12-12

**Authors:** Tianxiang Yang, Zhizhong Mi, Zhaolong Li

**Affiliations:** 1 Institute of Virology and AIDS Research, The First Hospital of Jilin University, Changchun, Jilin, China; 2 Department of Infectious Diseases, Infectious Diseases and Pathogen Biology Center, Key Laboratory of Organ Regeneration and Transplantation of The Ministry of Education, The First Hospital of Jilin University, Changchun, Jilin, China

**Keywords:** caspase-8, immune amplification, multimodal cell death, NLRP3 inflammasome, regulated cell death, RSV, therapeutic targeting

## Abstract

Respiratory syncytial virus (RSV) is a major cause of severe respiratory tract infections in infants, older adults, and immunocompromised individuals. Despite decades of research, effective therapies are limited, largely due to an incomplete understanding of how infected cells and immune responses interact to shape disease outcomes. Recent evidence indicates that RSV activates multiple regulated cell death (RCD) programs-including apoptosis, necroptosis, pyroptosis, ferroptosis, and autophagy-associated cell death which interact through shared molecular mediators to form a multimodal cell death (MMCD) network. This integrated system regulates the balance between viral clearance and immunopathological injury. Central mediators such as caspase-8, RIPK3, and NLRP3 act as molecular hubs coordinating these death programs and amplifying inflammatory responses. Understanding how MMCD shapes RSV immunopathogenesis provides a unified framework linking cell death to immune dysfunction. This review summarizes recent progress in elucidating the MMCD network, highlights its role in death-inflammation feedback loops, and discusses potential strategies for therapeutic modulation. Conceptualizing RSV disease through the lens of MMCD may guide the development of precision interventions that restore immune homeostasis while preserving antiviral defense.

## 1 Introduction

RSV, a negative-sense RNA virus of the *Pneumovirus* genus (*Paramyxoviridae* family), is the leading cause of acute lower respiratory tract infections (ALRIs) in infants and young children worldwide. It also contributes significantly to pneumonia and respiratory failure among older adults and immunocompromised populations. Epidemiological data estimate that RSV infects more than 33 million children under five years of age each year, leading to over 100,000 deaths-most occurring in low- and middle-income countries (1). In older adults, RSV-associated hospitalization and mortality rates are comparable to or even exceed those of seasonal influenza (2), highlighting its growing cross-age public health significance.

At the mechanistic level, RSV infection is characterized by epithelial barrier disruption, mucus hypersecretion, and massive immune cell infiltration within the airway mucosa (3). Viral entry, mediated by the fusion (F) and attachment (G) glycoproteins, leads to syncytium formation. This process also triggers inflammatory signaling through the TLR4-NF-κB axis (4). These viral protein-host interactions generate inflammatory signaling and cellular stress-including oxidative imbalance, endoplasmic reticulum (ER) stress, and mitochondrial dysfunction that act as upstream triggers for RCD pathways. Emerging evidence suggests that this complex immunopathology is underpinned by a coordinated network of RCD pathways that collectively shape viral clearance, inflammation, and tissue remodeling (5). Historically, RSV-induced cell death was attributed mainly to apoptosis (6). However, advances in single-cell transcriptomics and spatial multi-omics have revealed that RSV simultaneously engages multiple RCD programs-apoptosis, pyroptosis, necroptosis, ferroptosis, and autophagy-related death-within the same tissue microenvironment (7, 8). These pathways are interconnected via shared molecular hubs such as caspase-8, RIPK3 (receptor-interacting serine/threonine-protein kinase 3), and NLRP3 (NOD-like receptor family pyrin domain-containing 3), forming a coordinated MMCD network. Despite accumulating evidence, the mechanisms by which MMCD orchestrates immune dysregulation and tissue injury during RSV infection remain unclear. This mini review summarizes current knowledge of MMCD, its immunopathological implications, and its potential as a target for precision intervention.

## 2 Molecular basis of cell death signaling during RSV infection

The RSV genome is a single-stranded, negative-sense RNA approximately 15.2 kb in length, encoding 11 viral proteins. Among these, the fusion (F) and attachment (G) glycoproteins are central to viral entry and early host signaling. The F protein undergoes proteolytic activation by host enzymes, exposing the fusion peptide that mediates membrane merging and syncytium formation (9). This process simultaneously triggers the TLR4-MAPK-NF-κB axis, promoting the release of proinflammatory cytokines such as IL-6, TNF-α, and CXCL8 (C-X-C motif chemokine ligand 8) (10). The G protein, which contains a CX3C chemokine motif mimicking the host ligand CX3CL1 (C-X3-C motif chemokine ligand 1), binds to CX3CR1 (C-X3-C motif chemokine receptor 1) on epithelial and dendritic cells (11). This interaction facilitates viral attachment while transiently dampening immune cell recruitment and dendritic cell maturation (12), creating an initial phase of immune suppression. G protein-induced calcium dysregulation and oxidative stress further prime infected cells for downstream activation of RCD signaling.

The nonstructural proteins NS1 and NS2 function as potent antagonists of host antiviral defenses (13). The NS1/2 complex inhibits MAVS-mediated RIG-I-IRF3 signaling and promotes STAT2 degradation, thereby suppressing type I interferon responses (14). In addition, NS2 interferes with mitochondrial dynamics by preventing DRP1 (Dynamin-Related Protein 1) phosphorylation, leading to excessive ROS accumulation (15). The resulting oxidative stress activates ER stress-CHOP pathway, downregulates Bcl-2, and increases mitochondrial membrane permeability (16), predisposing cells to apoptosis, necroptosis, or ferroptosis under sustained infection.

Host receptors further contribute to RSV propagation and the integration of death signaling. CX3CR1, IGF1R (Insulin-like growth factor 1 receptor), and P2Y2 (P2Y purinoceptor 2) receptors facilitate viral entry and propagation (17, 18). IGF1R activation transiently delays apoptosis through the PI3K-AKT pathway, while P2Y2-mediated calcium influx promotes viral release. Prolonged ER stress through the PERK-eIF2α-CHOP axis amplifies mitochondrial dysfunction, providing a convergence point for apoptotic, necroptotic, and ferroptotic pathways. Together, these viral and host interactions lay the molecular foundation for the integrated MMCD network that underpins RSV pathogenesis.

## 3 Forms of regulated cell death in RSV infection

**Figure 1:**
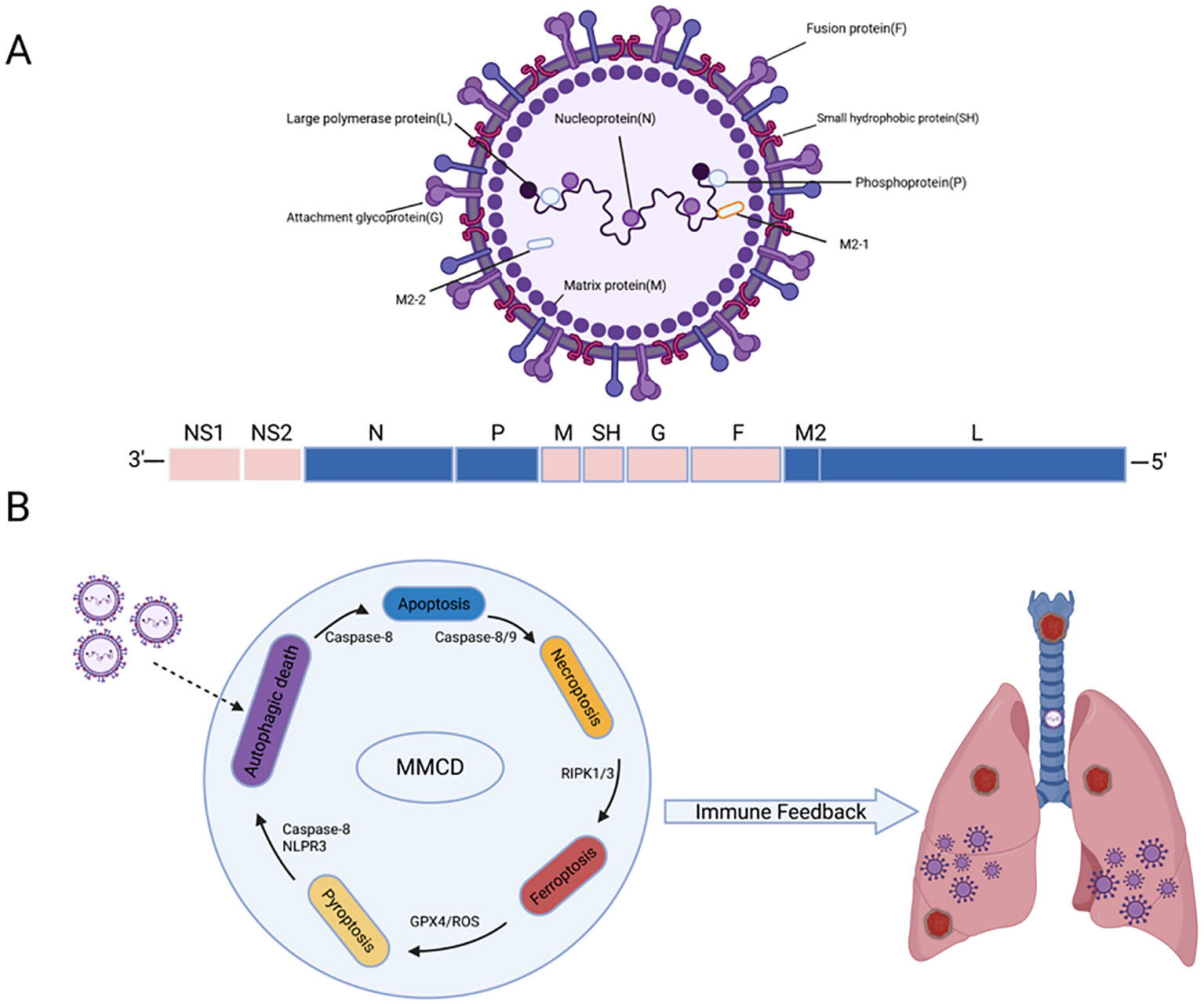
Overview of RSV structure and the integrated MMCD network. **(A)** Schematic representation of the RSV virion showing surface glycoproteins (F, G, SH), matrix (M), and nucleocapsid components (N-P-L-M2 complex), as well as regulatory proteins NS1/NS2. **(B)** Viral invasion triggers multiple regulated cell death pathways within infected airway epithelial cells. Caspase-8 acts as a molecular switch linking apoptosis and necroptosis through Caspase-8/9 and RIPK1/3 signaling. Pyroptosis is driven by Caspase-8-NLRP3 activation, while ferroptosis is mediated by GPX4 inhibition and ROS accumulation. Autophagic cell death contributes additional stress signals. These interconnected pathways form an integrated MMCD feedback loop at the core of this circuit lies the Caspase-8-RIPK3-NLRP3 molecular kernel, amplifying oxidative stress and inflammatory signaling. Thus, the resulting immune feedback promotes airway injury and immunopathogenesis in the infected lung.

RSV infection activates multiple RCD programs that operate in a coordinated and context-dependent manner (**Figure 1**), rather than acting independently, apoptosis, pyroptosis, necroptosis, ferroptosis, and autophagy-related death intersect through shared molecular hubs-including caspase-8, RIPK3, NLRP3, and GPX4 (Glutathione Peroxidase 4) that determine whether infected cells undergo containment, inflammatory amplification, or oxidative injury.

During early infection, controlled apoptosis and autophagy contribute to viral clearance while limiting inflammation. As infection progresses, inflammatory RCD pathways such as pyroptosis and necroptosis dominate, releasing DAMPs (Damage-associated molecular patterns) that amplify cytokine production and epithelial injury. In later stages, ferroptosis-driven oxidative stress promotes chronic epithelial dysfunction and tissue remodeling. Viral proteins (F, G, NS1/2) and host stress responses cooperatively regulate these transitions through mitochondrial, ER, and lipid metabolic pathways.

**Figure 1** depicts the integrated framework in which RSV components trigger signaling cascades that converge on the MMCD network, producing overlapping waves of regulated cell death (RCD) that collectively shape immune pathogenesis. **Table 1** summarizes key regulators of these pathways and their corresponding immunopathological outcomes. Together, these findings highlight that RSV pathogenicity stems not from a single mode of cell death, but from the hierarchical integration of multiple death programs that determine the magnitude of inflammation, the failure of tissue repair, and overall disease severity.

**Table 1:** Representative regulators and immunopathological consequences of RCD during RSV infection.

RCD category	Representative regulators	Pathophysiological role	References
Early containment (apoptosis, autophagy)	Caspase-3/8/9, Bcl-2, LC3-II, Beclin-1	Viral clearance, limitation of early inflammation	(19, 20)
Inflammatory amplification (pyroptosis, necroptosis)	NLRP3, ASC, GSDMD, RIPK1/3, MLKL	Cytokine release, epithelial rupture, immune cell recruitment	(21–24)
Oxidative injury (ferroptosis)	GPX4, Nrf2, ROS, Fe^2+^	Lipid peroxidation, mitochondrial dysfunction, chronic inflammation	(25, 26)

## 4 Integrative network and immunopathological coupling

The MMCD network is governed by a central regulatory triad-the Caspase-8-RIPK3-NLRP3 molecular kernel-which serves as the signaling core that balances apoptosis, necroptosis, and pyroptosis. This molecular kernel integrates stress and death cues, ensuring the dynamic coordination of multimodal cell death responses during RSV infection. Under physiological conditions, caspase-8 restrains necroptosis by cleaving RIPK1 and RIPK3. During RSV infection, NS1/2 proteins inhibit caspase-8 activation and promote RIPK3-MLKL assembly, redirecting apoptotic signals toward necroptotic and pyroptotic outputs (27, 28). This triad-based network has also been implicated in SARS-CoV-2 and influenza pathogenesis, underscoring its conserved immunoregulatory role across viral infections. The ensuing potassium efflux and mitochondrial ROS generation further activate NLRP3, establishing a feed-forward inflammatory loop that amplifies tissue injury.

Beyond intracellular regulation, the MMCD network tightly interacts with innate and adaptive immune circuits. DAMPs and alarmins released from necroptotic and pyroptotic cells stimulate dendritic cells and macrophages via TLR4 and RAGE, fueling IL-1β, IL-6, and TNF-α production (29). These cytokines, in turn, promote Th17 polarization and neutrophil recruitment, which reinforce oxidative stress and perpetuate ferroptotic signaling (30, 31). Spatial transcriptomics has revealed that epithelial, myeloid, and stromal cell subsets engage distinct MMCD modules within inflamed lung tissue (32). This spatial integration underscores MMCD as a systems-level mechanism linking cellular fate to immune orchestration.

Together, the Caspase-8-RIPK3-NLRP3 triad functions as the molecular kernel of MMCD, integrating apoptotic, necroptotic, pyroptotic, and ferroptotic signals into a unified immunopathological continuum. This framework redefines RSV-induced injury as a dynamic equilibrium between protective elimination and destructive amplification.

## 5 Feedback loops between cell death and immune activation

MMCD not only results from infection but also actively shapes immune responses. Distinct forms of RCD release a spectrum of intracellular contents-DAMPs, alarmins, and oxidized lipids-that engage pattern recognition receptors (PRRs) on immune cells. These outputs are orchestrated by the Caspase-8-RIPK3-NLRP3 molecular kernel, which couples regulated cell death to immune amplification. Molecules such as HMGB1, ATP, and mitochondrial DNA activate macrophages and dendritic cells via TLR4, NLRP3, and RAGE, triggering cytokine cascades dominated by IL-1β, IL-6, TNF-α, and type I interferons (33, 34). These cytokines further sensitize epithelial and immune cells to RCD activation by upregulating death receptors such as Fas and TRAILR.

Conversely, sustained immune activation feeds back to intensify cellular stress pathways. Persistent production of IFN-γ and TNF-α induces ER stress, mitochondrial dysfunction, and oxidative damage, shifting the balance from controlled apoptosis to necroptosis and ferroptosis (35). Activated neutrophils and Th17 cells release reactive oxygen and nitrogen species, exacerbating lipid peroxidation and impairing tissue repair. This bidirectional crosstalk establishes a “death-inflammation” loop, in which MMCD-derived signals perpetuate immune activation, and immune effector molecules in turn reinforce multimodal cell death.

Beyond acute infection, MMCD-driven inflammation influences long-term immune remodeling. Repeated epithelial injury and chronic oxidative stress reshape the airway microenvironment, leading to aberrant macrophage polarization and exhaustion of tissue-resident memory T cells (36, 37). Such maladaptive remodeling may contribute to post-RSV wheezing and asthma-like sequelae observed in susceptible individuals. Understanding these feedback dynamics provides insight into how transient infection evolves into chronic immunopathology. Such maladaptive remodeling may explain post-viral airway hyperresponsiveness and provide a mechanistic bridge between infection and chronic respiratory disorders.

Collectively, MMCD acts as both a driver and target of immune regulation, forming a dynamic feedback network that connects cellular fate with immune homeostasis. Dissecting this loop could uncover novel biomarkers for disease severity and identify intervention points where selective modulation of MMCD may break the cycle of inflammation and tissue injury, setting the MMCD as both an effector and regulator of immune homeostasis, warranting integrative therapeutic exploration.

## 6 Discussion

The concept of MMCD provides a unified framework to interpret the immunopathogenesis of RSV infection. By integrating apoptosis, pyroptosis, necroptosis, ferroptosis, and autophagy-related processes into a coordinated signaling continuum, MMCD explains how the same virus can simultaneously elicit antiviral defense and excessive inflammation. The hierarchical organization of MMCD-ranging from early containment to chronic oxidative injury-clarifies the dynamic balance between protective clearance and destructive immunopathology. This integrative view expands beyond conventional single-pathway models and highlights the Caspase-8-RIPK3-NLRP3 axis as a pivotal regulatory triad governing immune amplification.

From a translational perspective, the MMCD paradigm opens new opportunities for precision immunotherapy. Translating MMCD insights into therapy demands identification of druggable nodes within this integrated network. Pharmacological inhibition of RIPK1, NLRP3, or ferroptosis regulators (e.g. Necrostatin-1, MCC950, Deferoxamine) has shown promise in preclinical models by mitigating epithelial cell necrosis and restoring immune balance (38, 39). Rather than complete suppression, selective modulation of MMCD pathways may recalibrate host responses without compromising antiviral immunity. Moreover, profiling MMCD-associated signatures through single-cell multi-omics or spatial transcriptomics could enable patient stratification and the identification of biomarkers predicting disease severity or treatment response.

Nevertheless, several challenges remain. First, the heterogeneity of MMCD responses across cell types and disease stages complicates therapeutic targeting. Second, pharmacodynamic optimization and delivery barriers limit clinical translation of existing inhibitors. Third, the dual role of MMCD in both defense and pathology raises concerns that broad inhibition may inadvertently impair antiviral immunity. Future studies should therefore adopt context-specific modulation approaches guided by quantitative modeling and *in vivo* validation. For instance, early-phase inhibition of the NLRP3 inflammasome might suppress excessive epithelial or macrophage pyroptosis and reduce IL-1β-mediated inflammation-a notion supported by recent studies showing amelioration of RSV-induced immunopathology upon NLRP3 blockade (40). In more severe or late-stage disease characterized by widespread necroptosis and cell lysis, targeting RIPK1/RIPK3 or downstream MLKL may prevent necroptotic amplification, consistent with observations of necroptosis involvement in RSV-infected macrophages (41).Integrating MMCD dynamics into systems immunology frameworks could reveal emergent properties that predict therapeutic windows and adverse outcomes.

Current evidence connecting individual MMCD pathways to RSV pathogenesis is largely correlative, as most mechanistic insights are derived from *in vitro* experiments or animal models rather than human tissues. Establishing true causality will require longitudinal clinical sampling, single-cell-resolved analyses, and functional perturbation studies in physiologically relevant systems. Moreover, although conceptual temporal models-such as “early apoptotic containment” versus “late-stage inflammatory necroptosis or pyroptosis”- provide a useful framework, MMCD programs *in vivo* are likely to operate simultaneously and are strongly influenced by cell type, tissue organization, metabolic context, and local immune cues. This inherent spatiotemporal complexity poses challenges for precise *in vivo* validation. Finally, therapeutic targeting of MMCD remains constrained by issues of specificity. Intervening in one pathway may unintentionally impair beneficial antiviral immunity or provoke compensatory death programs. A critical challenge in the therapeutic targeting of necroptosis lies in achieving sufficient pathway specificity while minimizing off-target effects. Kinase inhibitors, particularly those acting on RIPK1 or RIPK3, may exhibit unintended interactions with other signaling kinases, leading to undesirable side effects. Moreover, inhibiting necroptosis can inadvertently redirect cell death signaling toward alternative programmed pathways, such as apoptosis or pyroptosis, due to compensatory network activation. These complexities underscore the need for developing next-generation inhibitors with improved selectivity, temporal control, and context-dependent modulation to ensure therapeutic efficacy and safety.

Future research should therefore focus on dissecting pathway cross-talk and designing compounds that maintain the delicate balance between inhibition and cellular adaptation. Thus, effective clinical translation will require strategies that enable cell-type-selective or spatially restricted modulation of detrimental MMCD signaling while preserving essential host defense mechanisms.

In summary, viewing RSV immunopathogenesis through the lens of MMCD transforms our understanding of virus-host interactions from a linear cascade into an interconnected, self-regulating network. This conceptual shift emphasizes that immunopathology arises not from isolated molecular events but from the dynamic orchestration of multimodal cell death and immune feedback. Elucidating and therapeutically harnessing this network may ultimately pave the way for precision interventions that disrupt immunopathology while preserving host defense in RSV and other viral inflammatory diseases.

## 7 Methods

We performed a literature search in PubMed, Web of Science using the keywords “RSV”, “RCD”, “autophagy”, “necroptosis”, “pyroptosis”, “ferroptosis”, “NLRP3”, “RIPK3”,”MMCD”and “immunopathogenesis”. Articles published between 2015–2025 were included. Additional references were identified through manual screening of relevant reviews and cross-referencing of primary research articles.
